# Supplementary material for: Incineration Kinetic Analysis of Upstream Oily Sludge and Sectionalized Modeling in Differential/Integral Method
Source: Int J Environ Res Public Health. 2019 Jan 29;16(3):384. doi: 10.3390/ijerph16030384 (PMC6388273; doi:10.3390/ijerph16030384)
Supplement: Supplementary file 1 [file ijerph-16-00384-s001.pdf]

International Journal of
Environmental Research and Public Health

MDPI

$$P(\mu) = \int_{\infty}^{\mu} -e^{-\mu}\,\mu^{-2} d\mu$$

$$= \int_{\infty}^{\mu} \mu^{-2}\, de^{-\mu} = \frac{e^{-\mu}}{\mu^2}\,|_{\infty}^{\mu} - \int_{\infty}^{\mu} e^{-\mu}\, d\mu^{-2}$$

$$= \frac{e^{-\mu}}{\mu^2}\,|_{\infty}^{\mu} - \int_{\infty}^{\mu} e^{-\mu}\,(-2)\mu^{-3} d\mu$$

$$= \frac{e^{-\mu}}{\mu^2} - 2\int_{\infty}^{\mu} \mu^{-3}\, de^{-\mu}$$

$$= \frac{e^{-\mu}}{\mu^2} - 2\frac{e^{-\mu}}{\mu^3} + \int_{\infty}^{\mu} e^{-\mu}\,(-6)\mu^{-4} d\mu$$

$$= \frac{e^{-\mu}}{\mu^2} - 2\frac{e^{-\mu}}{\mu^3} + 6\frac{e^{-\mu}}{\mu^4} - \int_{\infty}^{\mu} 24e^{-\mu}\,\mu^{-5} d\mu$$

$$= \frac{e^{-\mu}}{\mu^2} - 2\frac{e^{-\mu}}{\mu^3} + 6\frac{e^{-\mu}}{\mu^4} - 24\frac{e^{-\mu}}{\mu^5} + \int_{\infty}^{\mu} 24e^{-\mu}\, d\mu^{-5}$$

$$= \frac{e^{-\mu}}{\mu^2}\left(1 - \frac{2!}{\mu} + \frac{3!}{\mu^2} - \frac{4!}{\mu^3}\cdots\right)$$

$$= \frac{e^{-\mu}}{\mu^2} \times \sum_{n=1}^{\infty} (-1)^{n-1} \frac{n!}{\mu^{n-1}}$$

Figure 1. Solution of P(μ).

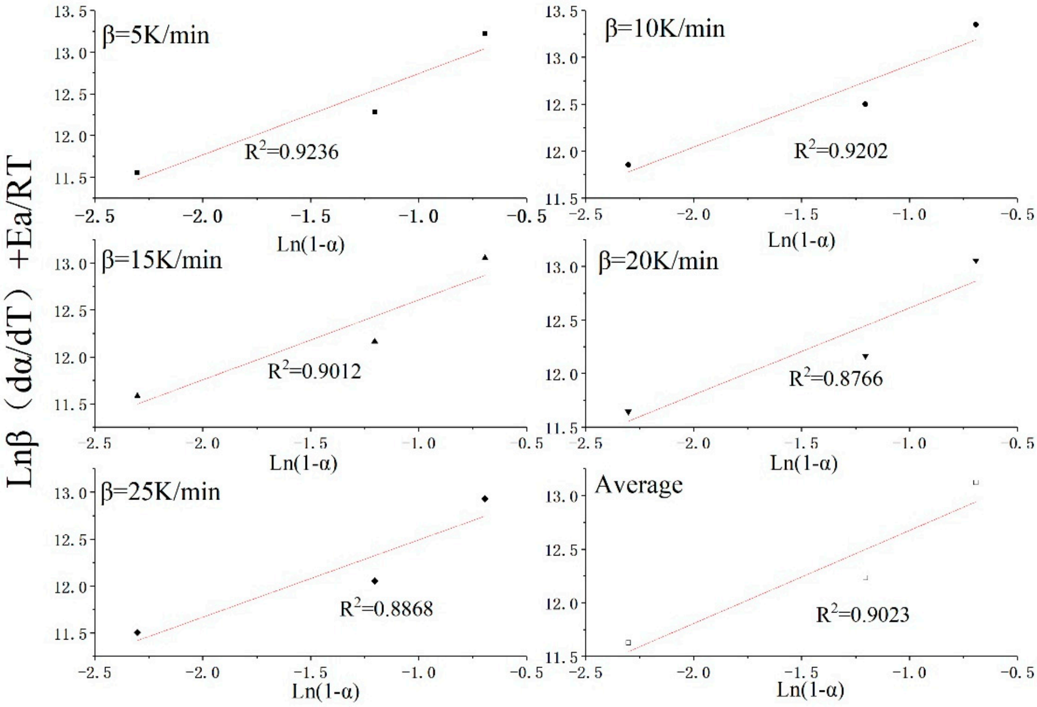

Figure 2. The linear fitting results for reaction orders and pre-exponential factors of stage one.
